# Supplementary material for: Causal Relationship between Aging and Anorexia Nervosa: A White-Matter-Microstructure-Mediated Mendelian Randomization Analysis
Source: Biomedicines. 2024 Aug 16;12(8):1874. doi: 10.3390/biomedicines12081874 (PMC11351342; doi:10.3390/biomedicines12081874)
Supplement: Supplementary file 1 [file biomedicines-12-01874-s001.zip › Supplementary Figure/Figure S4/Singe SNP plot of MR analysis for TL to ubm-b-2098.pdf]

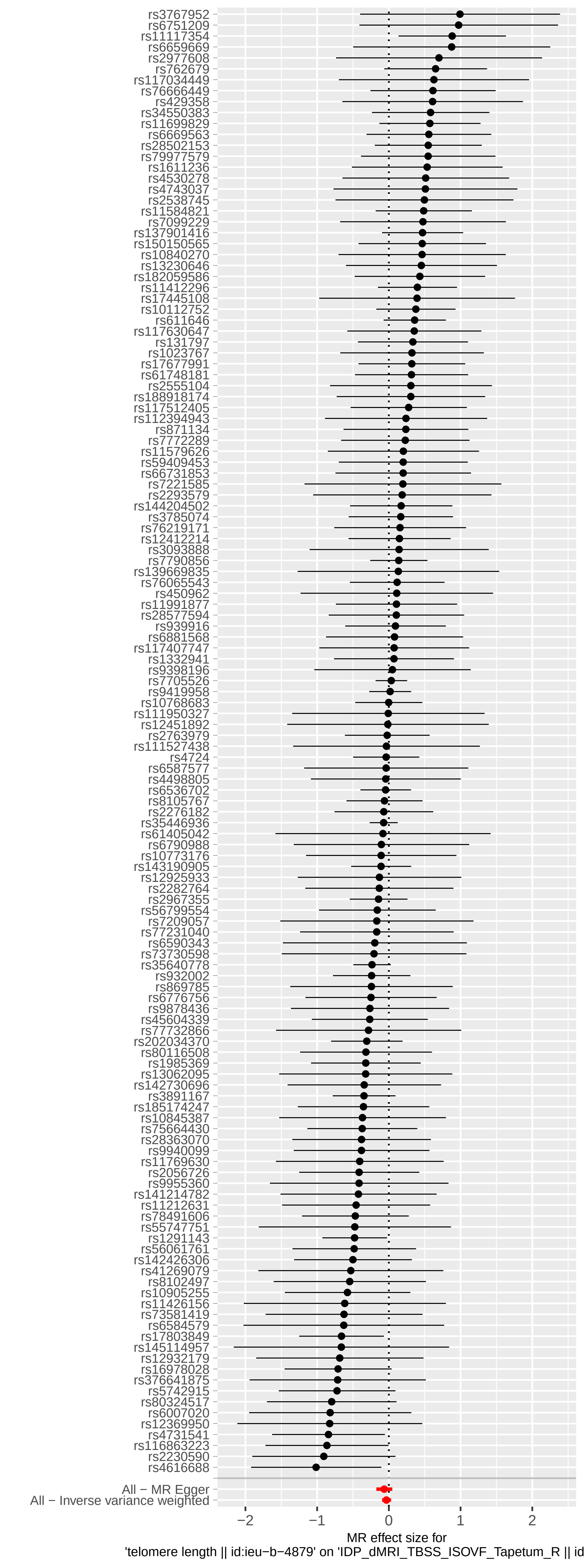
rs3767952
rs6751209
rs11117354
rs6659669
rs2977608
rs762679
rs117034449
rs76666449
rs429358
rs34550383
rs11699829
rs6669563
rs28502153
rs79977579
rs1611236
rs4530278
rs4743037
rs2538745
rs11584821
rs7099229
rs137901416
rs150150565
rs10840270
rs13230646
rs182059586
rs11412296
rs17445108
rs10112752
rs611646
rs117630647
rs131797
rs1023767
rs17677991
rs61748181
rs2555104
rs188918174
rs117512405
rs112394943
rs871134
rs7772289
rs11579626
rs59409453
rs66731853
rs7221585
rs2293579
rs144204502
rs3785074
rs76219171
rs12412214
rs3093888
rs7790856
rs139669835
rs76065543
rs450962
rs11991877
rs28577594
rs939916
rs6881568
rs117407747
rs1332941
rs9398196
rs7705526
rs9419958
rs10768683
rs111950327
rs12451892
rs2763979
rs111527438
rs4724
rs6587577
rs4498805
rs6536702
rs8105767
rs2276182
rs35446936
rs61405042
rs6790988
rs10773176
rs143190905
rs12925933
rs2282764
rs2967355
rs56799554
rs7209057
rs77231040
rs6590343
rs73730598
rs35640778
rs932002
rs869785
rs6776756
rs9878436
rs45604339
rs77732866
rs202034370
rs80116508
rs1985369
rs13062095
rs142730696
rs3891167
rs185174247
rs10845387
rs75664430
rs28363070
rs9940099
rs11769630
rs2056726
rs9955360
rs141214782
rs11212631
rs78491606
rs55747751
rs1291143
rs56061761
rs142426306
rs41269079
rs8102497
rs10905255
rs11426156
rs73581419
rs6584579
rs17803849
rs145114957
rs12932179
rs16978028
rs376641875
rs5742915
rs80324517
rs6007020
rs12369950
rs4731541
rs116863223
rs2230590
rs4616688
All − MR Egger
All − Inverse variance weighted
−2
−1
0
1
2
MR effect size for
'telomere length || id:ieu−b−4879' on 'IDP_dMRI_TBSS_ISOVF_Tapetum_R || id
